# Comprehensive analysis of the glutathione S-transferase Mu (GSTM) gene family in ovarian cancer identifies prognostic and expression significance

**DOI:** 10.3389/fonc.2022.968547

**Published:** 2022-07-28

**Authors:** Juan Zhang, Yan Li, Juan Zou, Chun-tian Lai, Tian Zeng, Juan Peng, Wen-da Zou, Bei Cao, Dan Liu, Li-yu Zhu, Hui Li, Yu-kun Li

**Affiliations:** ^1^ Department of Assisted Reproductive Centre, Zhuzhou central hospital, Xiangya hospital Zhuzhou central south university, Central south university, Zhuzhou, China; ^2^ Hunan Province Key Laboratory of Tumor Cellular and Molecular Pathology, Cancer Research Institute, University of South China, Hengyang, China

**Keywords:** ovarian cancer, bioinformatic analysis, GSTM family, prognostic marker, drug sensitivity

## Abstract

**Background:**

Ovarian cancer (OC) is one of the most common types of gynecologic tumor over the world. The Glutathione S-transferase Mu (GSTM) has five members, including GSTM1-5. These GSTMs is involved in cell metabolism and detoxification, but their role in OC remains unknown.

**Methods:**

Data from multiple public databases associated with OC and GSTMs were collected. Expression, prognosis, function enrichment, immune infiltration, stemness index, and drug sensitivity analysis was utilized to identify the roles of GSTMs in OC progression. RT-qPCR analysis confirmed the effect of AICAR, AT-7519, PHA-793887 and PI-103 on the mRNA levels of GSTM3/4.

**Results:**

GSTM1-5 were decreased in OC samples compared to normal ovary samples. GSTM1/5 were positively correlated with OC prognosis, but GSTM3 was negatively correlated with OC prognosis. Function enrichment analysis indicated GSTMs were involved in glutathione metabolism, drug metabolism, and drug resistance. Immune infiltration analysis indicated GSTM2/3/4 promoted immune escape in OC. GSTM5 was significantly correlated with OC stemness index. GSTM3/4 were remarkedly associated with OC chemoresistance, especially in AICAR, AT-7519, PHA-793887 and PI-103.

**Conclusion:**

GSTM3 was negatively correlated with OC prognosis, and associated with OC chemoresistance and immune escape. This gene may serve as potential prognostic biomarkers and therapeutic target for OC patients.

## Introduction

Ovarian cancer (OC), a serious obstetrical and gynecological malignant disease, ranks eighth in terms of morbidity and mortality overall, resulting in huge economic and health problems (1). About 70 percent of OC patients diagnosed at an advanced stage develop metastases that result in a loss of surgery, due to the absence of early detection strategies and symptoms (2). For most OC patients, traditional chemotherapy has extremely high side effects and poor efficacy (3). Therefore, it is urgent to find effective diagnostic markers and therapeutic targets for ovarian cancer.

Glutathione S-transferase Mu (GSTM) gene family is group of 5 proteins, GSTM1-5, that play a key role in the detoxification of electrophilic compounds, such as cancer-causing toxins, anticarcinogens and products of oxidative stress *via* conjugating with glutathione (4). The catalytic activities of these GSTMs can repress pK_a_ of the sulfhydryl group of reduced glutathione (GSH) from 9.0 in aqueous solution to about 6.5 when GSH is bound in the active site (5). These GSTM proteins are increased during drug treatment, resulting in chemotherapy resistance (6). Moreover, the highly polymorphic, and allele mutations or genetic deletions of a certain base of GSTMs enhance the predisposition for multiple cancer, such as colon cancer (7), cervical cancer (8), esophageal cancer (9), lung cancer (10), and acute myeloid leukaemia (11). For example, GSTM1 was a high-polymorphically expressed gene, which was confirmed three alleles, including GSTM1-0, GSTM1a, and GSTM1b. The inactivation of GSTM by homozygous delegation was unable to efficiently estimate these electrophilic compounds. Nevertheless, there has no epidemiologic studies to find the correlation between GSTM1 and OC (12). Therefore, more evidence is needed on the biological functions, prognostic and diagnostic significance of the GSTMs for OC development and progression.

**Figure 1:**
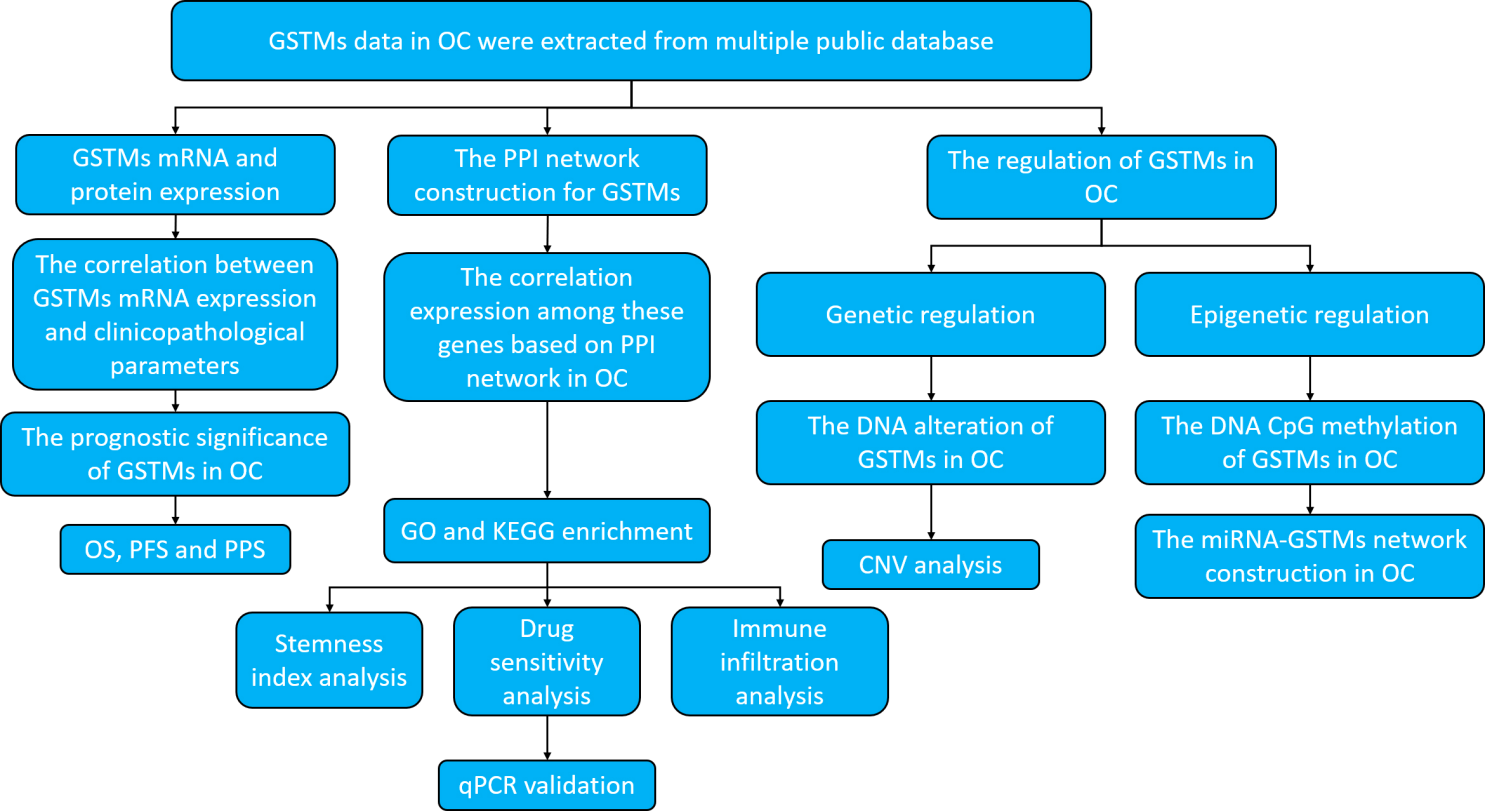
Work flow of the study.

For clearly elucidate GSTM gene family in OC, especially in prognostic and expression significance. we utilized multiple public databases to elucidate the role of GSTMs in OC progression, including the DNA alteration, mRNA and protein expression, epigenetic regulations, biological functions, molecular interactions, and signaling pathways enrichments. The research strategy is showed in **Figure 1**.

## Methods

### Bioinformatic expression analysis

Oncomine database (https://www.oncomine.org) (13) and the cancer genome atlas (TCGA) (https://www.cancer.gov/tcga) (14) were used to prepare the expression data for GSTM1-5 mRNA expression level in OC patients. Clinical Proteomic Tumor Analysis Consortium (CPTAC) database (https://proteomics.cancer.gov/programs/cptac) (15) was utilized to confirm the GSTM1-5 protein expression level in OC patients. Human protein atlas (HPA) database (https://www.proteinatlas.org/) (16) was used to confirm the protein level of GSTMs in OC. The patient’s information based on HPA database was illustrated in **Supplementary Table 1**. The expression of GSTM1-5 in multiple OC cell lines was used Cancer Cell Line Encyclopedia (CCLE) database (https://sites.broadinstitute.org/ccle) (17). GTEX database (https://www.gtexportal.org/) was used to prepare the expression data for GSTM1-5 mRNA expression level in normal ovarian tissue samples (18).

### DNA alteration analysis

The cBioPortal database (http://www.cbioportal.org/) (19) was used to confirm GSTM1-5 alteration and survival outcome for OC patients.

### Protein structure analysis of GSTMs

Protein Data Bank (PDB) (https://www.rcsb.org/) (20) was used to analyze secondary structure of GSTM1-5.

### Survival analysis

We analyzed these five GSTM family genes, GSTM1, GSTM2, GSTM3, GSTM4 and GSTM5 by Kaplan Meier plotter (KM-plot) database (http://kmplot.com/) (21) in OC patients. We used the best threshold be a cutoff, which were based the all feasible cutoff between the upper and lower quartiles.

### Protein-protein interaction (PPI) network construction

GeneMANIA 3.6.0 (http://www.genemania.org) (22) was utilized to constructed the PPI network associated with GSTM1, GSTM2, GSTM3, GSTM4 and GSTM5.

### Gene ontology and Kyoto encyclopedia of genes and genomes enrichment analysis

Database for Annotation, Visualization and Integrated Discovery (DAVID) database (https://david.ncifcrf.gov/) (23) was used for GO and KEGG enrichment analysis for these correlated GSTM1-5 genes based on the PPI network.

### Immune infiltration analysis

RNA-sequencing profiles and corresponding clinical information for OC were extracted from the TCGA database. We utilized immuneeconv, an R software package that integrates six latest algorithms (TIMER, xCell, MCP-counter, CIBERSORT, EPIC and quanTIseq), to analysis the reliable results of immune score. SIGLEC15, TIGIT, CD274, HAVCR2, PDCD1, CTLA4, LAG3 and PDCD1LG2 were selected to be immune-checkpoint-relevant transcripts and the expression values of these eight genes were extracted. Immune infiltration analysis for Copy number variations (CNV) of GSTMs was used by Tumor Immune Estimation Resource (TIMER) database (https://cistrome.shinyapps.io/timer/) (24).

### Stemness index analysis

Use the one-class logistic regression machine learning (OCLR) algorithm to calculate mRNAsi which constructed by Malta et al (25). We utilized the same Spearman correlation (RNA expression data). The minimum value was subtracted, and the result was divided by the maximum maps the dryness index to the range [0,1] based on TCGA database.

### Drug sensitivity analysis

Gene Set Cancer Analysis (GSCA) database (http://bioinfo.life.hust.edu.cn/GSCA/#/) (26), an friendly interacted and integrated public database, was used to make the drug sensitivity analysis for GSTM1-5.

### Cell culture

OC cell lines, Hey-A8 (purchased from ATCC), was cultured in RPMI−1640 medium (Thermo Fisher Scientific,Inc.) with 10% (v/v) fetal bovine serum (FBS; Gibco; Invitrogen; Thermo Fisher Scientific, Inc.) and 1% Penicillin-Streptomycin mixture (Thermo Fisher Scientific, Inc.). The Hey-A8 was treatment with AICAR (2 mM), AT-7519 (40 nM), PHA-793887 (1 μM) and PI-103 (50 nM) for 48 h at 37˚C, respectively. These chemical compounds were purchased from Abmole Bioscience Inc.

### RT-qPCR analysis

The RT-qPCR assay was executed as illustrated previously (27). Primers used were listed as followed: GAPDH forward: GTCTCCTCTGACTTCAACAGCG, GAPDH reverse: ACCACCCTGTTGCTGTAGCCAA; GSTM3 forward: CGAAGCCAATGGCTGGATGTGA, GSTM3 reverse: GTTGTGCTTGCGAGCGATGTAG; GSTM4 forward: TGGAGAACCAGGCTATGGACGT, GSTM4 reverse: CCAGGAACTGTGAGAAGTGCTG;

### Statistical analysis

All statistical analyses were based on the R Programming Language (version 3.6). For immune infiltration, immune score, drug sensitivity, and stemness index analysis, the statistical difference of two groups was compared through the Wilcox test, significance difference of three groups was tested with Kruskal-Wallis test. The KM-plot survival analysis with log-rank test were also used to compare the survival difference between above two groups. The Student’s t-tests analysis was used to compare the GSTM3/4 mRNA level difference in RT-qPCR analysis. P-values <0.05 were considered significant.

## Results

### The mRNA and protein expression of GSTMs in OC

**Figure 2:**
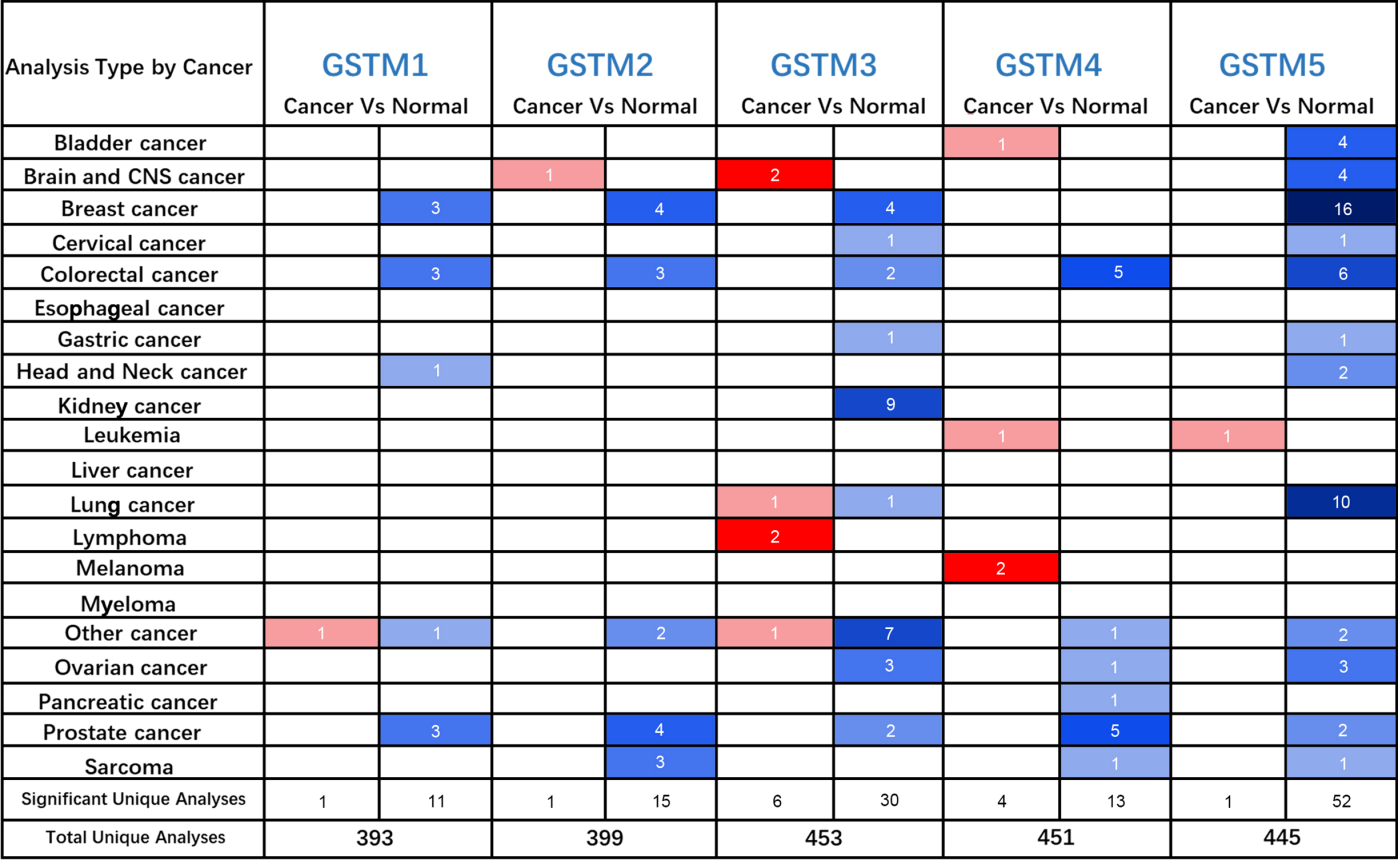
The GSTMs mRNA level in multiple cancer types. The red color cell indicates that GSTMs is enhanced in tumor samples compared to correspond normal samples, whereas blue color cell presents GSTMs is reduced in tumor samples compared to correspond normal samples.

**Figure 3:**
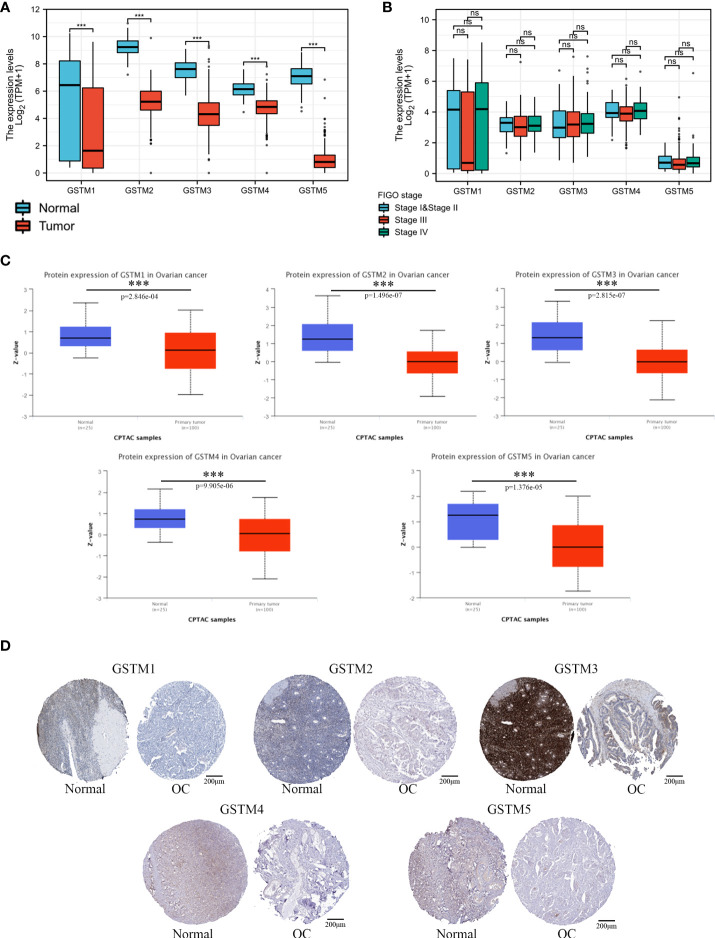
The GSTMs expression in OC. **(A)** The mRNA level of GSTMs in OC samples and normal ovary samples based on TCGA and GETx database. **(B)** The mRNA level of GSTMs in different FIGO stage OC samples. **(C)** The protein level of GSTMs in OC samples and normal ovary samples based on CPTAC database. **(D)** The GSTM protein expression in OC samples and normal ovary samples based on HPA database. ***p < 0.001 ns, means No statistical significance.

Firstly, we used the Oncomine database to confirm GSTMs mRNA level in multiple cancer types compared to the corresponding para-carcinoma samples (**Figure 2**), which showed that GSTM1/2/3/4/5 were significantly decreased in many cancers, especially in OC. In the total unique analyses, GSTM1 was 393 datasets; GSTM2 was 399 datasets; GSTM3 was 453 datasets; GSTM4 was 451 datasets; GSTM5 was 445 datasets. Moreover, GSTM3/4/5 were significantly decreased in 30, 13 and 52 datasets, respectively. We further confirmed the level of GSTMs in OC compared to normal ovarian tissue samples based on TCGA database (**Figure 3A**), indicating that GSTMs mRNA were both significantly decreased in OC tissue samples. However, the level of these GSTMs mRNA were not present statistic difference among different stages (**Figure 3B**). We further detected the GSTM1-5 proteins in OC samples and normal ovarian samples, which showed that both GSTM1-5 were decreased in OC patients comparted to normal women (**Figure 3C**). Moreover, the result of GSTMs protein expression in OC based on HPA database was consistent with CPTAC database (**Figure 3D**). These results indicated that the transcription and post-transcription levels of GSTMs were both decreased in OC patients.

### The potential regulatory mechanisms of GSTMs in OC

In order to further clarify the cause of ectopic expression of GSTMs in OC, we analyzed the m5C methylation level of GSTMs in CpG island of DNA promoter, as shown in **Supplementary Figure 1A**. The DNA methylation of GSTM1/5 was significantly increased in OC compared to normal samples, but the DNA methylation of GSTM3 and GSTM4 was significantly decreased in OC compared to normal samples. Not only that, the miRNA network showed that GSTM2/3/4/5 was markedly regulated by multiple miRNAs, such as hsa-miR-455-5p, hsa-miR-142-5p, hsa-miR-377-3p, hsa-miR-939-5p and so on (**Supplementary Figure 1B**). These results indicated that epigenetic regulation play a key role in the ectopic expression of GSTMs.

### The correlation between GSTMs and clinicopathological parameter for OC patients

Subsequently, we also confirmed the correlation between GSTMs and clinicopathological parameter in OC patients based on TCGA database, as shown in **Table 1**–**5**. GSTM1 was significantly associated with histologic grade and race. GSTM2 was obviously correlated with race, age and venous invasion. GSTM4 levels associated with race and venous invasion. But the expression of GSTM3 and GSTM5 were not remarkedly correlated with any clinicopathological parameters in OC patients. Taken together, these results indicated that these clinicopathological parameters might be involved in the ectopic expression of GSTMs in OC development, especially in age and race.

**Table 1:** The correlation between pathological parameters and GSTM1 expression.

Characteristic	Low expression of GSTM1	High expression of GSTM1	p
n	189	190	
FIGO stage, n (%)			0.353
Stage I	1 (0.3%)	0 (0%)	
Stage II	10 (2.7%)	13 (3.5%)	
Stage III	153 (40.7%)	142 (37.8%)	
Stage IV	24 (6.4%)	33 (8.8%)	
Primary therapy outcome, n (%)			0.482
PD	13 (4.2%)	14 (4.5%)	
SD	13 (4.2%)	9 (2.9%)	
PR	17 (5.5%)	26 (8.4%)	
CR	107 (34.7%)	109 (35.4%)	
Race, n (%)			0.004
Asian	3 (0.8%)	9 (2.5%)	
Black or African American	6 (1.6%)	19 (5.2%)	
White	174 (47.7%)	154 (42.2%)	
Age, n (%)			0.873
<=60	105 (27.7%)	103 (27.2%)	
>60	84 (22.2%)	87 (23%)	
Histologic grade, n (%)			0.039
G1	0 (0%)	1 (0.3%)	
G2	29 (7.9%)	16 (4.3%)	
G3	154 (41.7%)	168 (45.5%)	
G4	1 (0.3%)	0 (0%)	
Anatomic neoplasm subdivision, n (%)			0.277
Unilateral	56 (15.7%)	46 (12.9%)	
Bilateral	122 (34.2%)	133 (37.3%)	
Venous invasion, n (%)			0.177
No	18 (17.1%)	23 (21.9%)	
Yes	38 (36.2%)	26 (24.8%)	
Lymphatic invasion, n (%)			0.730
No	23 (15.4%)	25 (16.8%)	
Yes	53 (35.6%)	48 (32.2%)	
Tumor residual, n (%)			0.935
NRD	34 (10.1%)	33 (9.9%)	
RD	132 (39.4%)	136 (40.6%)	
Tumor status, n (%)			0.711
Tumor free	38 (11.3%)	34 (10.1%)	
With tumor	131 (38.9%)	134 (39.8%)	
Age, meidan (IQR)	58 (50, 67)	59 (52, 68)	0.357

**Table 2:** The correlation between pathological parameters and GSTM2 expression.

Characteristic	Low expression of GSTM2	High expression of GSTM2	p
n	189	190	
FIGO stage, n (%)			0.285
Stage I	1 (0.3%)	0 (0%)	
Stage II	8 (2.1%)	15 (4%)	
Stage III	152 (40.4%)	143 (38%)	
Stage IV	27 (7.2%)	30 (8%)	
Primary therapy outcome, n (%)			0.123
PD	14 (4.5%)	13 (4.2%)	
SD	6 (1.9%)	16 (5.2%)	
PR	25 (8.1%)	18 (5.8%)	
CR	110 (35.7%)	106 (34.4%)	
Race, n (%)			0.044
Asian	4 (1.1%)	8 (2.2%)	
Black or African American	7 (1.9%)	18 (4.9%)	
White	168 (46%)	160 (43.8%)	
Age, n (%)			0.015
<=60	116 (30.6%)	92 (24.3%)	
>60	73 (19.3%)	98 (25.9%)	
Histologic grade, n (%)			0.873
G1	0 (0%)	1 (0.3%)	
G2	22 (6%)	23 (6.2%)	
G3	164 (44.4%)	158 (42.8%)	
G4	1 (0.3%)	0 (0%)	
Anatomic neoplasm subdivision, n (%)			0.933
Unilateral	52 (14.6%)	50 (14%)	
Bilateral	127 (35.6%)	128 (35.9%)	
Venous invasion, n (%)			0.035
No	13 (12.4%)	28 (26.7%)	
Yes	35 (33.3%)	29 (27.6%)	
Lymphatic invasion, n (%)			0.471
No	20 (13.4%)	28 (18.8%)	
Yes	50 (33.6%)	51 (34.2%)	
Tumor residual, n (%)			0.096
NRD	27 (8.1%)	40 (11.9%)	
RD	141 (42.1%)	127 (37.9%)	
Tumor status, n (%)			1.000
Tumor free	36 (10.7%)	36 (10.7%)	
With tumor	131 (38.9%)	134 (39.8%)	
Age, meidan (IQR)	57 (48, 66)	61 (53, 71)	0.001

**Table 3:** The correlation between pathological parameters and GSTM3 expression.

Characteristic	Low expression of GSTM3	High expression of GSTM3	p
n	189	190	
FIGO stage, n (%)			1.000
Stage I	1 (0.3%)	0 (0%)	
Stage II	12 (3.2%)	11 (2.9%)	
Stage III	147 (39.1%)	148 (39.4%)	
Stage IV	28 (7.4%)	29 (7.7%)	
Primary therapy outcome, n (%)			0.182
PD	16 (5.2%)	11 (3.6%)	
SD	7 (2.3%)	15 (4.9%)	
PR	19 (6.2%)	24 (7.8%)	
CR	113 (36.7%)	103 (33.4%)	
Race, n (%)			0.699
Asian	7 (1.9%)	5 (1.4%)	
Black or African American	11 (3%)	14 (3.8%)	
White	166 (45.5%)	162 (44.4%)	
Age, n (%)			0.324
<=60	109 (28.8%)	99 (26.1%)	
>60	80 (21.1%)	91 (24%)	
Histologic grade, n (%)			0.285
G1	0 (0%)	1 (0.3%)	
G2	19 (5.1%)	26 (7%)	
G3	164 (44.4%)	158 (42.8%)	
G4	0 (0%)	1 (0.3%)	
Anatomic neoplasm subdivision, n (%)			1.000
Unilateral	52 (14.6%)	50 (14%)	
Bilateral	129 (36.1%)	126 (35.3%)	
Venous invasion, n (%)			0.177
No	18 (17.1%)	23 (21.9%)	
Yes	38 (36.2%)	26 (24.8%)	
Lymphatic invasion, n (%)			0.133
No	21 (14.1%)	27 (18.1%)	
Yes	59 (39.6%)	42 (28.2%)	
Tumor residual, n (%)			0.495
NRD	30 (9%)	37 (11%)	
RD	135 (40.3%)	133 (39.7%)	
Tumor status, n (%)			0.641
Tumor free	33 (9.8%)	39 (11.6%)	
With tumor	132 (39.2%)	133 (39.5%)	
Age, meidan (IQR)	58 (49, 67)	60 (51, 71)	0.086

**Table 4:** The correlation between pathological parameters and GSTM4 expression.

Characteristic	Low expression of GSTM4	High expression of GSTM4	p
n	189	190	
FIGO stage, n (%)			0.092
Stage I	1 (0.3%)	0 (0%)	
Stage II	12 (3.2%)	11 (2.9%)	
Stage III	155 (41.2%)	140 (37.2%)	
Stage IV	21 (5.6%)	36 (9.6%)	
Primary therapy outcome, n (%)			0.998
PD	14 (4.5%)	13 (4.2%)	
SD	11 (3.6%)	11 (3.6%)	
PR	22 (7.1%)	21 (6.8%)	
CR	112 (36.4%)	104 (33.8%)	
Race, n (%)			0.020
Asian	7 (1.9%)	5 (1.4%)	
Black or African American	6 (1.6%)	19 (5.2%)	
White	172 (47.1%)	156 (42.7%)	
Age, n (%)			0.714
<=60	106 (28%)	102 (26.9%)	
>60	83 (21.9%)	88 (23.2%)	
Histologic grade, n (%)			0.056
G1	0 (0%)	1 (0.3%)	
G2	29 (7.9%)	16 (4.3%)	
G3	157 (42.5%)	165 (44.7%)	
G4	0 (0%)	1 (0.3%)	
Anatomic neoplasm subdivision, n (%)			0.750
Unilateral	53 (14.8%)	49 (13.7%)	
Bilateral	126 (35.3%)	129 (36.1%)	
Venous invasion, n (%)			0.046
No	16 (15.2%)	25 (23.8%)	
Yes	39 (37.1%)	25 (23.8%)	
Lymphatic invasion, n (%)			0.351
No	21 (14.1%)	27 (18.1%)	
Yes	54 (36.2%)	47 (31.5%)	
Tumor residual, n (%)			0.461
NRD	31 (9.3%)	36 (10.7%)	
RD	140 (41.8%)	128 (38.2%)	
Tumor status, n (%)			0.284
Tumor free	32 (9.5%)	40 (11.9%)	
With tumor	139 (41.2%)	126 (37.4%)	
Age, meidan (IQR)	58 (50, 68)	59 (52, 68)	0.345

**Table 5:** The correlation between pathological parameters and GSTM5 expression.

Characteristic	Low expression of GSTM5	High expression of GSTM5	p
n	189	190	
FIGO stage, n (%)			0.489
Stage I	1 (0.3%)	0 (0%)	
Stage II	10 (2.7%)	13 (3.5%)	
Stage III	151 (40.2%)	144 (38.3%)	
Stage IV	25 (6.6%)	32 (8.5%)	
Primary therapy outcome, n (%)			0.268
PD	12 (3.9%)	15 (4.9%)	
SD	9 (2.9%)	13 (4.2%)	
PR	18 (5.8%)	25 (8.1%)	
CR	118 (38.3%)	98 (31.8%)	
Race, n (%)			0.406
Asian	8 (2.2%)	4 (1.1%)	
Black or African American	14 (3.8%)	11 (3%)	
White	161 (44.1%)	167 (45.8%)	
Age, n (%)			0.233
<=60	110 (29%)	98 (25.9%)	
>60	79 (20.8%)	92 (24.3%)	
Histologic grade, n (%)			0.322
G1	0 (0%)	1 (0.3%)	
G2	19 (5.1%)	26 (7%)	
G3	164 (44.4%)	158 (42.8%)	
G4	1 (0.3%)	0 (0%)	
Anatomic neoplasm subdivision, n (%)			0.750
Unilateral	49 (13.7%)	53 (14.8%)	
Bilateral	129 (36.1%)	126 (35.3%)	
Venous invasion, n (%)			0.618
No	17 (16.2%)	24 (22.9%)	
Yes	31 (29.5%)	33 (31.4%)	
Lymphatic invasion, n (%)			1.000
No	23 (15.4%)	25 (16.8%)	
Yes	48 (32.2%)	53 (35.6%)	
Tumor residual, n (%)			1.000
NRD	33 (9.9%)	34 (10.1%)	
RD	133 (39.7%)	135 (40.3%)	
Tumor status, n (%)			0.669
Tumor free	38 (11.3%)	34 (10.1%)	
With tumor	130 (38.6%)	135 (40.1%)	
Age, meidan (IQR)	58 (51, 68)	60 (51, 67.75)	0.782

### The possible molecular functions of GSTMs in OC patients

**Figure 4:**
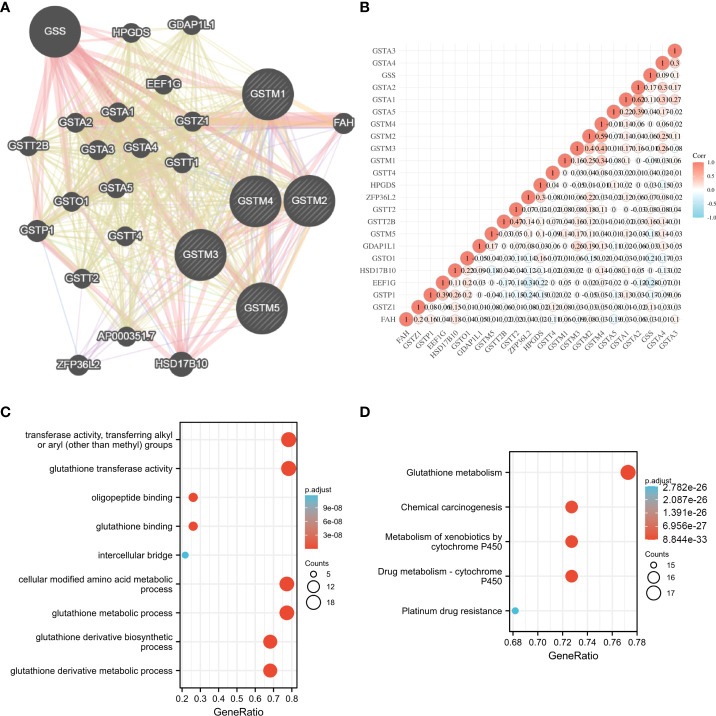
The potential molecular function of GSTMs in OC. **(A)** The PPI network associated with GSTM1-5 based on GeneMANIA database. **(B)** Correlation heat map of PPI network based on TCGA database. **(C)** The GO enrichment of PPI network genes. **(D)** The KEGG enrichment of PPI network genes.

To further explore the possible molecular functions of the GSTM proteins in OC development and progression, we constructed the PPI network for GSTM1-5 and relevant proteins based on GeneMANIA database, these relevant proteins included GSS, HPGDS, GDAP1L1, GSTT2B, GSTA2, GSTA1, EEF1G, GSTA3, GSTA4, GSTZ1, GSTP1, GSTO1, GSTA5, GSTT1, GSTT2, GSTT4, ZFP36L2, AP000351.7, HSD17B10 (**Figure 4A**). Moreover, we make a correlation analysis among these genes based on TCGA database OC dataset (**Figure 4B**). We also analyzed the GO enrichment for these genes. These genes were enriched in transferase activity, transferring alkyl or aryl (other than methyl) groups, glutathione transferase activity, oligopeptide binding, glutathione binding, intercellular bridge, cellular modified amino acid metabolic process, glutathione metabolic process, glutathione derivative biosynthetic process, and glutathione derivative metabolic process (**Figure 4C**). KEGG enrichment analysis showed that these genes were enriched in glutathione metabolism, chemical carcinogenesis, metabolism of xenobiotics by cytochrome P450, drug metabolism - cytochrome P450, and platinum drug resistance (**Figure 4D**). Furthermore, we detected the secondary structure of GSTM1-5 proteins, which indicated that both GSTMs had same domains, such as GST_N and GST_C domain. The secondary structure of GSTM proteins also suggested that phosphorylation and ubiquitination were the two main chemical modifications (**Supplementary Figure 2**).

### Prognostic value of GSTM family members for OC patients

**Figure 5:**
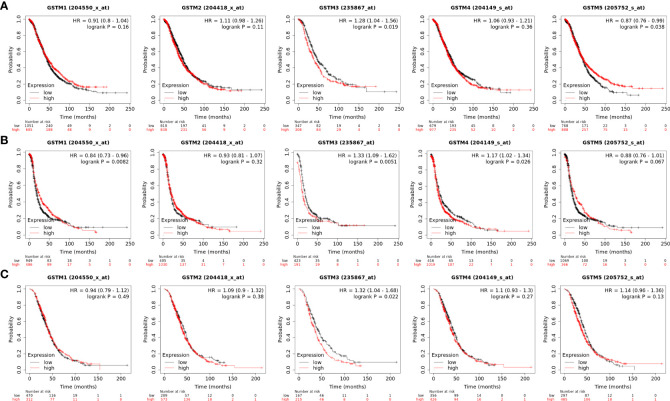
The prognosis significance of GSTM1-5 in OC. **(A)** The OS analysis of GSTM1/2/3/4/5 in OC dataset based on the Kaplan–Meier Plotter database. **(B)** The PFS analysis of GSTM1/2/3/4/5 in OC dataset based on the Kaplan–Meier Plotter database. **(C)** The PPS analysis of GSTM1/2/3/4/5 in OC dataset based on the Kaplan–Meier Plotter database.

Moreover, we extracted GSTM1-5 mRNA level data and prognostic data based on KM-plot database. The overall survival (OS) analysis showed that GSTM3 was negatively correlated with the prognosis of OC patients, but GSTM5 was positively correlated with the prognosis of OC patients (**Figure 5A**). The progression free survival (PFS) analysis showed that GSTM3/4 were negatively correlated with the prognosis of OC patients, but GSTM1 was positively correlated with the prognosis of OC patients (**Figure 5B**). The post progression survival (PPS) analysis showed that GSTM3 was negatively correlated with the prognosis of OC patients (**Figure 5C**). These results indicated that GSTM3 might be a significant prognostic marker for OC patients.

### The association between GSTMs and immune infiltration

**Figure 6:**
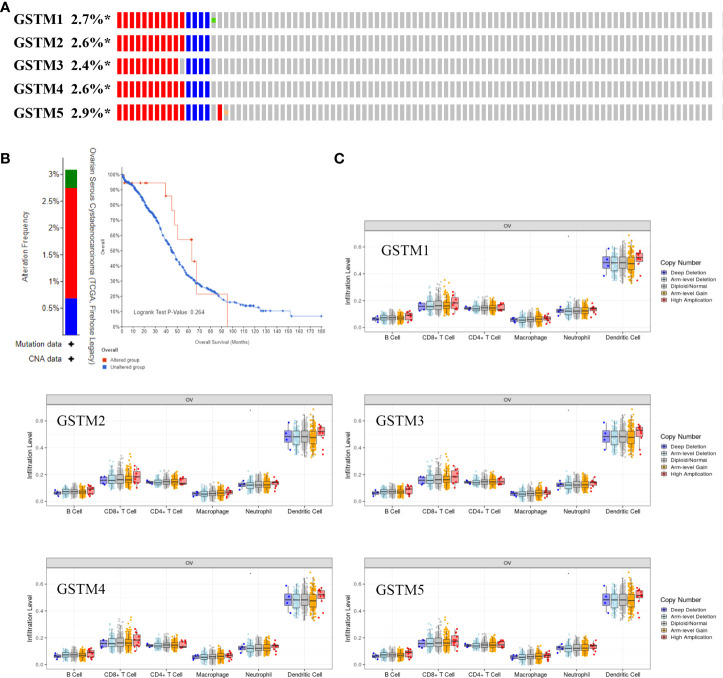
The correlation of immune infiltration and GSTMs alteration in OC. **(A)** The DNA alteration rate of GSTM1, GSTM2, GSTM3, GSTM4 and GSTM5 was more than 2% in OC. **(B)** The overall survival rate based on OC patients with and without these genes alteration based on the cBioPortal dabtase. **(C)** The effect of GSTM1-5 CNV on the immune cell distribution based on the TIMER database. *p < 0.05.

**Figure 7:**
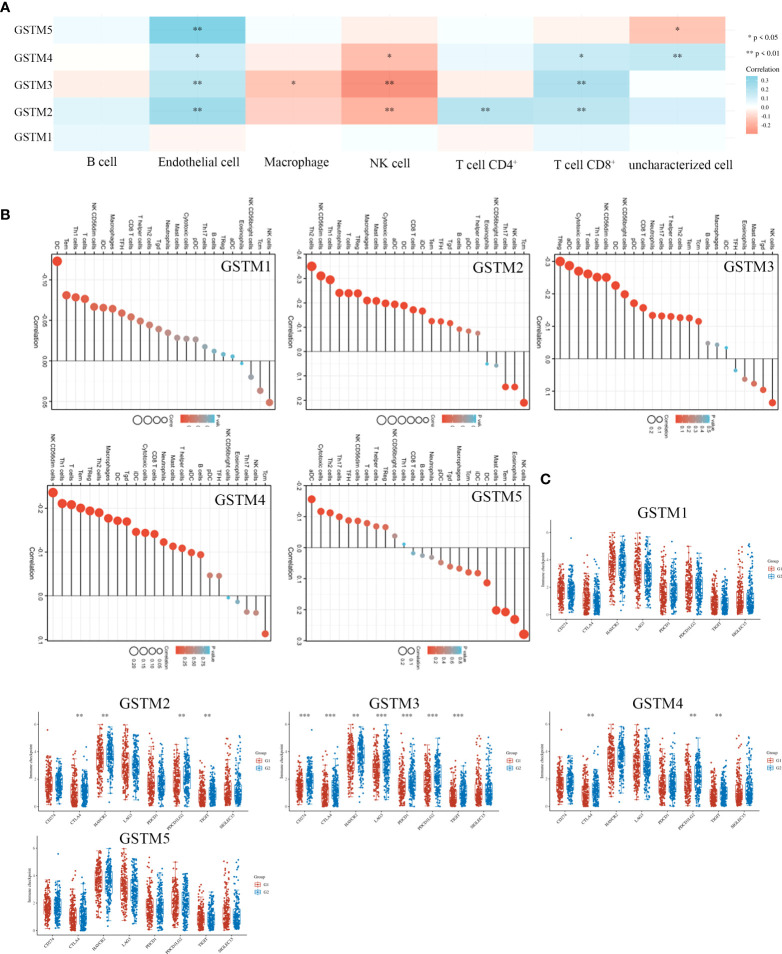
The correlation of immune infiltration and GSTMs expression in OC. **(A)** The expression profiles of GSTM1, GSTM2, GSTM3, GSTM4 and GSTM5 in multiple immune cell types based on TCGA database. **(B)** The effect of GSTMs on the components of cellular immunity. **(C)** The correlation analysis between GSTMs expression and immune checkpoints gene expression in TCGA database *via* the Wilcox test. (G1 is the group of the OC patients with high expression of GSTMs. G2 is the group of the OC patients with low expression of GSTMs.) *p < 0.05; **p < 0.01; ***p < 0.001.

Recently, immune infiltration is another hot point for OC treatment (28). Firstly, we extracted the DNA alteration profiles based on cBioProtal database OC dataset. The DNA alteration of GSTMs were 2.7%, 2.6%, 2.4%, 2.6% and 2.9%, respectively (**Figure 6A**). However, the DNA alteration of these GSTMs were not significantly correlated with prognosis or immune infiltration in patients with OC (**Figures 6B, C**). We further confirmed the correlation between GSTMs mRNA level and immune cell infiltration level. The result showed that GSTM2-5 were significantly and negatively associated with Endothelial cell; GSTM3 was positively correlated with macrophage; GSTM2-4 were significantly correlated with NK cells; the expression of GSTM2 was negatively associated with CD4+ T cell; GSTM2-4 were negatively associated with CD8+ T cell (**Figure 7A**). Furthermore, we also elucidate the effect of GSTMs on the components of cellular immunity, including aDC, B cells, CD8 T cells, Cytotoxic cells, DC, Eosinophils, iDC, Macrophages, Mast cells, Neutrophils, NK CD56bright cells, NK CD56dim cells, NK cells, pDC, T cells, T helper cells, Tcm, Tem, TFH, Tgd, Th1 cells, Th17 cells, Th2 cells and Treg by ssGSEA analysis (**Figure 7B**), which indicated that these GSTMs could regulate immune infiltration *via* inducing dysregulation of immune cell profiles. We further used CIBERSORT analysis to confirm the T cell features for high expression of GSTMs compared to corresponding low expression of GSTMs. The result suggested that GSTM1 regulated the level of B cell naive significantly (**Supplementary Figures 3A, B**); GSTM2 was obviously correlated with CD8 T cell (**Supplementary Figures 3C, D**); GSTM3 was associated with macrophoage M2, macrophoage M1, and T cell qamma delta (**Supplementary Figures 3E, F**); GSTM4 was correlated with macrophoage M2, CD8 T cell, and memory activated CD4 T cell (**Supplementary Figures 3G, H**). GSTM5 was correlated with macrophoage M0, and Mast cell activated (**Supplementary Figures 3I, J**). Moreover, we confirmed the expression level of immune checkpoints between high GSTMs mRNA level group and low GSTMs mRNA level group, as shown in **Figure 7C**. The result indicated that CTLA4, HAVCR3, PDCD1LG2 and TIGIT level were significantly decreased in high GSTM2 mRNA level group compared to low GSTM2 mRNA level group; the expression of TIGIT, CD274, HAVCR2, PDCD1, CTLA4, LAG3 and PDCD1LG2 were both markedly reduced in high GSTM3 mRNA level group compared to low GSTM3 mRNA level group; CTLA4, PDCD1LG2 and TIGIT expressions were obviously down-regulated in high GSTM4 mRNA level group compared to low GSTM4 mRNA level group.

### The effect of GSTM proteins on stemness in OC cell

**Figure 8:**
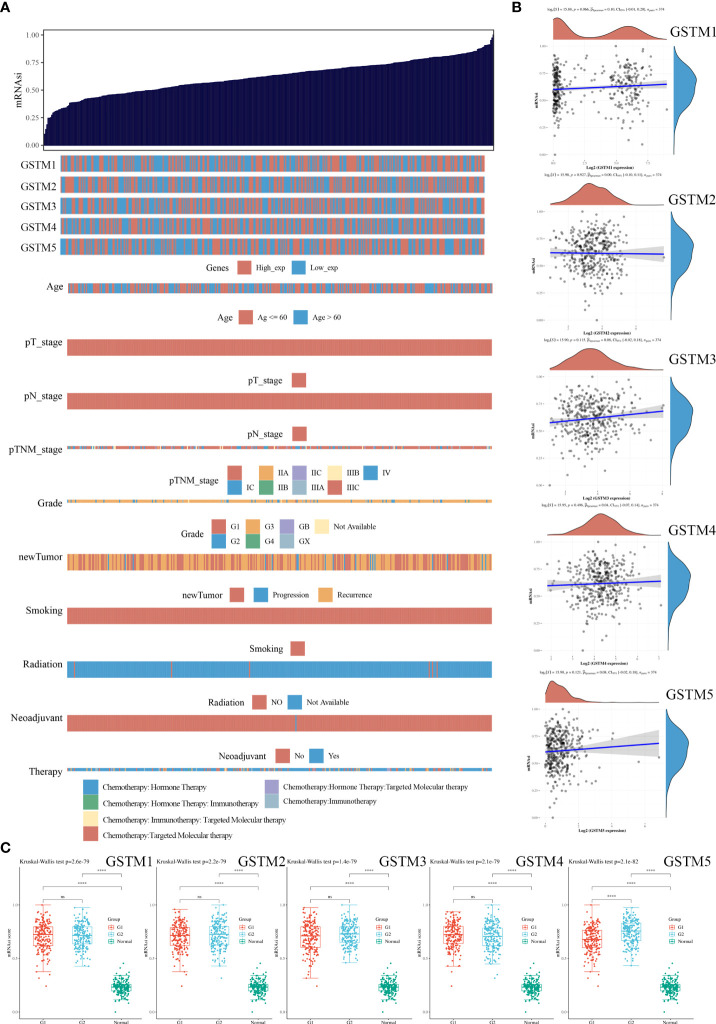
The association of stemness features and GSTMs in OC. **(A)** The stemness score heatmap of GSTM expression and clinical information. The top figure is the stemness score from low to high, and the bottom figure is the distribution of GSTMs expression and clinical information features after sorting. **(B)** Correlation analysis of stemness score and GSTMs gene expression. **(C)** The distribution of stemness scores in high expression of GSTMs OC groups, low expression of GSTMs OC groups, and normal ovary groups. (G1 is the group of the OC patients with high expression of GSTMs. G2 is the group of the OC patients with low expression of GSTMs.) ****p < 0.0001.

Since stemness features are the main causes of aberrant survival capacity and evasion of apoptosis during OC progression, we ranked the OC samples according to stemness index (from low to high) and tested whether any demographic/GSTMs expression level/clinical feature was associated with either a low or high stemness index (**Figure 8A**). Correlation analysis suggested that the total GSTMs mRNA level was not significantly correlated with stemness index in both OC patients (**Figure 8B**). Therefore, we further detected the difference of stemness index in high GSTMs level OC patients compared to low GSTMs level OC patients or normal women. The results indicated that the stemness index was only decreased in high GSTM5 level OC patients compared to low GSTM5 level OC patients. Moreover, the stemness index was significantly decreased in both normal ovary samples compared to OC tissues (**Figure 8C**). These results indicated that high expression of GSTM5 might possessed lower stemness features than OC patients with low expression of GSTM5.

### Determination of the drug sensitivity of the GSTMs

**Figure 9:**
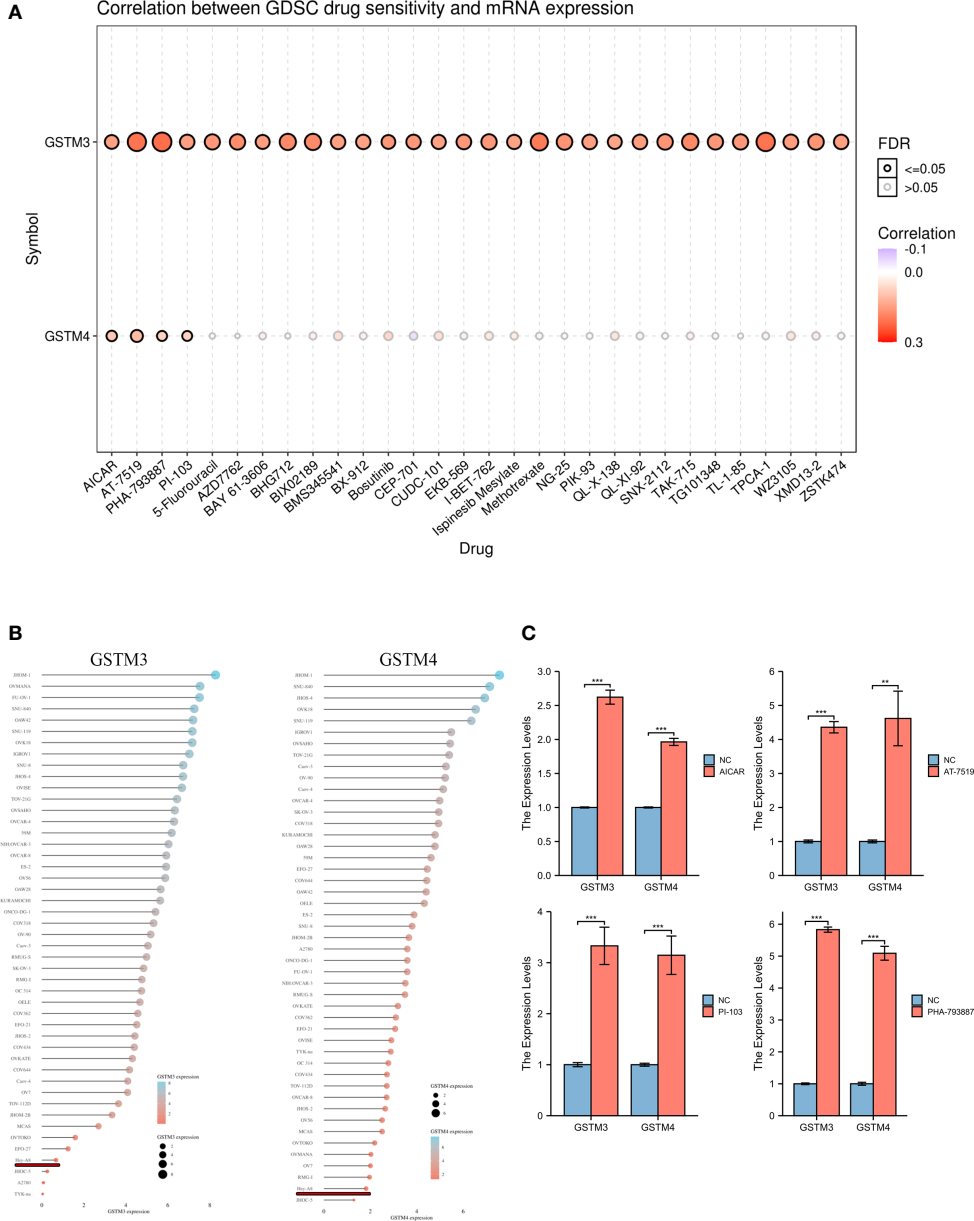
The Drug Sensitivity of GSTMs in OC. **(A)** The effect of multiple drugs on mRNA expression of GSTMs. **(B)** The mRNA expression of GSTM3/4 in multiple OC cell lines based on CCLE database. **(C)** The effects of AICAR, AT-7519, PHA-793887 and PI-103 on GSTM3/4 expression level in Hey-A8 cell lines. **p < 0.01; ***p < 0.001.

Finally, we confirmed the drug sensitivity of these GSTMs based on GSCA database (http://bioinfo.life.hust.edu.cn/GSCA/#/drug). The result showed that GSTM3/4 was upregulated in the treatment of multiple drugs, especially in AICAR, AT-7519, PHA-793887 and PI-103 (**Figure 9A**). For further select suitable cell lines for verification, we analyzed the GSTM3/4 level in OC cell lines based on CCLE database, as shown in **Figure 9B**. We chose Hey-A8 cell lines to detect the effect of **AICAR (2 mM), AT-7519 (40 nM), PHA-793887 (1 μM) and PI-103 (50 nM)** on GSTM3/4 level. The result showed that these drugs could significantly upregulate the level of both GSTM3 and GSTM4 (**Figure 9C**).

## Discussion

In our study, we firstly confirmed the transcription and post-transcription level of GSTM1-5 in OC patients based on Oncomine, TCGA, HPA and CPTAC database. We found levels of GSTM1-5 were significantly reduced in OC tissue samples. A previous study indicated that GSTM1-3 were significantly decreased in colon cancer tissue samples compared to normal tissues samples (29). Dysregulation of these GSTMs has been reported in multiple cancer types, such as head and neck cancer (30), colon cancer (31), leiomyoma (32), lung cancer (33), liver cancer (34), and prostate cancer (35). These results indicated that the ectopic expression of GSTMs might play key roles in OC occurrence, development and progression.

For further confirming the roles of GSTMs for OC progression, Clinical data correlation analysis indicated that GSTM1/2/4 were both markedly associated with age and race. In many previous study, GSTM1/2/4 has been drawn attention upon the correlation with the genetic risk for many types of cancers among multiple different races, such as Asian (36, 37), African (38), Northeast India (39), and European (40), which also partially explained why different ethnic groups have different cancer risk rates. The correlation between age and GSTM1/2/4 expression also suggested that the these GSTMs might be epigenetically regulated by environmental stimuli, including air, toxic chemicals, water, and radioactive rays. In previous studies, many researchers found that age was significantly correlated with GSTM1/2 in multiple diseases, including cataract (41), macular degeneration (42), essential hypertension (43), breast cancer (44), Parkinson’s disease (45), and ovarian damage (46). Moreover, we found GSTM2/4 were associated with venous invasion in OC patients, which indicated that GSTM2/4 might regulate the occurrence and development of venous invasion. Han et al. found that GSTM2 was involved in the regulation of other survival genes to promote proliferation and angiogenesis progression in ovarian teratoma (47). Noni Extract could regulate GSTM2 expression to impeded angiogenesis and proliferation in prostate cancer patients (48).

In order to further explore the molecular functions of GSTMs, GO and KEGG enrichment analysis based on PPI network showed that GSTM1-5 has a significantly effect on glutathione transferase activity, glutathione metabolism, chemical carcinogenesis, drug metabolism-cytochrome P450, and platinum drug resistance. These results indicated GSTM1-5 might be a performer to promote detoxification and the catabolism of electrophilic compounds *via* conjugating with glutathione. Sarhanis P et al. found that GSTM1 played a key role in the detoxification of the products of oxidative stress produced which induced the continued expression of the mutant protein during the repair of the ovarian epithelium, such as p53 mutation (49). GSTM1 also promoted cancer chemotherapy drugs or apoptosis escaping pathways to induce chemoresistance in liver cancer (34). Butyrate could significantly enhance GSTM2 level to act chemo-protectively by increasing detoxification capabilities in the colon mucosa (7). Peng et al. found that GSTM2 could upregulate chemotherapy resistance for gemcitabine in pancreatic cancer (50). GSTM3 polymorphism was a significant risk factor, and its expression was negatively correlated with disease free survival in esophageal squamous cell carcinoma (9, 51). Zhuo et al found that GSTM4 could repress etoposide-induced JNK activation and apoptosis (52). Luo and his colleagues also found that EWS/FLI bind to the promoter directly, which resulted in the increased expression of GSTM4 by GGAA-microsatellite in its promoter. GSTM4 deficiency inhibited the progression of Ewing’s sarcoma and chemotherapy resistance (53). Liu et al. indicated that CircRNA_0084927 sponged miRNA-20b-3p to increase the GSTM5 mRNA level, resulting in the transformation, development and progression of colorectal cancer (54). Taken together, these results indicated that GSTM proteins were promote cancer progression and chemoresistance *via* increasing detoxification capabilities and drug metabolism.

Moreover, we found GSTM3/4 expression were correlated with the poor prognosis, but GSTM3/4 mRNA and protein level were decreased in OC samples compared to normal ovary samples. The contradictory results might attribute to that the functions of GSTMs, especially in GSTM3/4, was induce detoxification of electrophilic compounds, such as cancer-causing toxins, anticarcinogens and products of oxidative stress *via* conjugating with glutathione (4). In the cancer initiation phase, the normal physiological functions of the normal cells were disturbed with the expression of GSTMs decreased, resulting in lower detoxification, which induce the development of cancer (55, 56). Then, the molecular function of GSTMs was protect cell from external stimuli in the development and progression of cancer, including anticarcinogens (57), which induced the development of drug resistance and the poor prognosis in cancer patients (58).

Moreover, we found GSTMs was significantly correlated with immune cell infiltration, GSTM2-4 were negatively associated with CD8 T cell. Li Y et al. found that GSTM2 could inhibit oxidative stress-induced renal cell apoptosis and inflammation in anti-glomerular basement membrane antibody-induced glomerulonephritis (59). Ren and his colleagues also found that GSTM3, as an antioxidant gene signature, to regulate immune cell infiltration and to predict the prognosis in patients with kidney renal clear cell carcinoma (60). Low expression of GSTM2/4 decrease ROS metabolism to induce Immunologic dysfunction in type 1 diabetes (61). This is probably due to the fact that GSTMs can mediate the dysfunction of immune cell function and composition by regulating the oxidative balance in the cell microenvironment.

OC stemness is a crucial role in metastasis and chemotherapy resistance (62). In this study, we found GSTM5 play a key role in OC cell stemness, which indicated GSTM5 might reduce ROS level to ameliorate oxidative stress, and ultimately, regulate OC stemness maintenance. The properties of stem cell mediate OC cells to avoid clinical standard chemotherapy, resulting in recurrent disease (63). Strong oxidative stress in OC cancer cells plays a hub role in mediating stemness maintenance, abnormal DNA replication, angiogenesis, lymphoangiogenesis, tumor microenvironment and metabolic reprogramming, all of which have been confirmed in chemotherapy resistance of OC (64). However, the correlation between OC stemness and GSTMs induced anti-oxidative stress remains unclarity.

Finally, we found GSTM3/4 expression were significantly correlated with multiple anti-cancer drugs, especially in AICAR, AT7519, PHA-793887 and PI-103. AICAR treatment inhibited the cell proliferation ability and spheroid formation of OC cells by activating AMPKα pathway, leading to downregulation of proliferation, stemness, and metastasis (65). AT7519, a cyclin-dependent kinase inhibitor, could significantly augments the efficacy of cisplatin *via* CDK, EMT, and apoptosis signaling (66). PHA-793887, as a potent CDK inhibitor, has an antiproliferative activity on OC cell *in vivo* and *in vitro (* 67). The inhibitor of class I phosphatidylinositide 3-kinases, PI-103, could decrease the chemotherapy resistance of the SKOV3/DDP OC cell line to cisplatin *in vitro* with pronounced antitumor efficacy (68). In our study, we found these anti-cancer drugs could significantly enhance the level of GSTM3/4 by a negative feedback way, resulting in promoting drug metabolism, especially in AICAR, AT-7519, PHA-793887 and PI-103. Combined with the results of multiple survival analysis, such as overall survival and meta-survival, GSTM3/4 could augment drug metabolism to blunt the effects of chemotherapy drugs, leading to poor prognosis for OC patients.

## Conclusion

In this study, we found both GSTM1-5 expressions were significantly downregulated in OC tissues compared to normal ovary tissues. GSTM3 was correlated with the OC poor prognosis, including OS, PFS and PPS. GSTM5 was positively correlated with the OC favorable prognosis, especially in OS. GSTM1 was associated with favorable prognosis in PFS. GSTM4 was associated with poor PFS in OC patients. Moreover, these GSTMs played key roles in immune infiltration of OC. GSTM5 might be involved in OC stemness features for OC cell. Drug sensitivity analysis indicated that AICAR, AT-7519, PHA-793887 and PI-103 increased GSTM3/4 expression to induce chemotherapy resistance. Therefore, our results can be a preliminary evidence for GSTM3 as a possible therapeutic target and prognostic marker for OC. Nevertheless, further work would be required to verify these candidates.

## Data availability statement

Publicly available datasets were analyzed in this study. This data can be found here: These data was analyzed based on mutiple public database, such as The Oncomine database (https://www.oncomine.org), the Cancer Genome Atlas (TCGA) (https://www.cancer.gov/tcga), The Clinical Proteomic Tumor Analysis Consortium (CPTAC) database (https://proteomics.cancer.gov/programs/cptac), The Cancer Cell Line Encyclopedia (CCLE) database (https://sites.broadinstitute.org/ccle), The cBioPortal database (http://www.cbioportal.org/), The Protein Data Bank (PDB) (https://www.rcsb.org/), The Kaplan–Meier plotter (KM-plot) database (http://kmplot.com/), GeneMANIA 3.6.0 (http://www.genemania.org), The Database for Annotation, Visualization and Integrated Discovery (DAVID) database (https://david.ncifcrf.gov/), and The Gene Set Cancer Analysis (GSCA) database (http://bioinfo.life.hust.edu.cn/GSCA/#/).

## Author contributions

JZ, YL, and CTL analyzed the data. TZ, JP and JZou used online tools. WDZ, BC and DL designed the project. LYZ selected the analyzed results. JZ wrote the paper. HL and YKL revised the manuscript, designed the experiment. All authors contributed to the article and approved the submitted version.

## Conflict of interest

The authors declare that the research was conducted in the absence of any commercial or financial relationships that could be construed as a potential conflict of interest.

## Publisher’s note

All claims expressed in this article are solely those of the authors and do not necessarily represent those of their affiliated organizations, or those of the publisher, the editors and the reviewers. Any product that may be evaluated in this article, or claim that may be made by its manufacturer, is not guaranteed or endorsed by the publisher.

## Supplementary material

Supplementary Figure 1 The regulatory mechanisms of GSTM members. **(A)** The DNA m5C methylation level of GSTM1-5 in OC patients and normal women. **(B)** The miRNA network of GSTMs based on GSCALite database.

Supplementary Figure 2 The protein structure of GSTM members.

Supplementary Figure 3 The expression distribution of GSTM1-5 immune score in OC samples and normal ovary samples. Immune cell score heatmap for GSTM1 **(A)**, GSTM2 **(C)**, GSTM3 **(E)**, GSTM4 **(G)** and GSTM5 **(I)** based on TCGA database *via* the Wilcox test. The percentage abundance of OC infiltrating immune cells in GSTM1 **(B)**, GSTM2 **(D)**, GSTM3 **(F)**, GSTM4 **(H)** and GSTM5 **(J)** group. Different colors indicated different immune cells types. The abscissa represents the OC sample, and the ordinate represents the percentage of immune cell content in each OC sample. *p < 0.05; **p < 0.01; ***p < 0.001.
