# Supplementary figures and images for: Comprehensive analysis of the glutathione S-transferase Mu (GSTM) gene family in ovarian cancer identifies prognostic and expression significance
Source: Front Oncol. 2022 Jul 28;12:968547. doi: 10.3389/fonc.2022.968547 (PMC9366399; doi:10.3389/fonc.2022.968547)

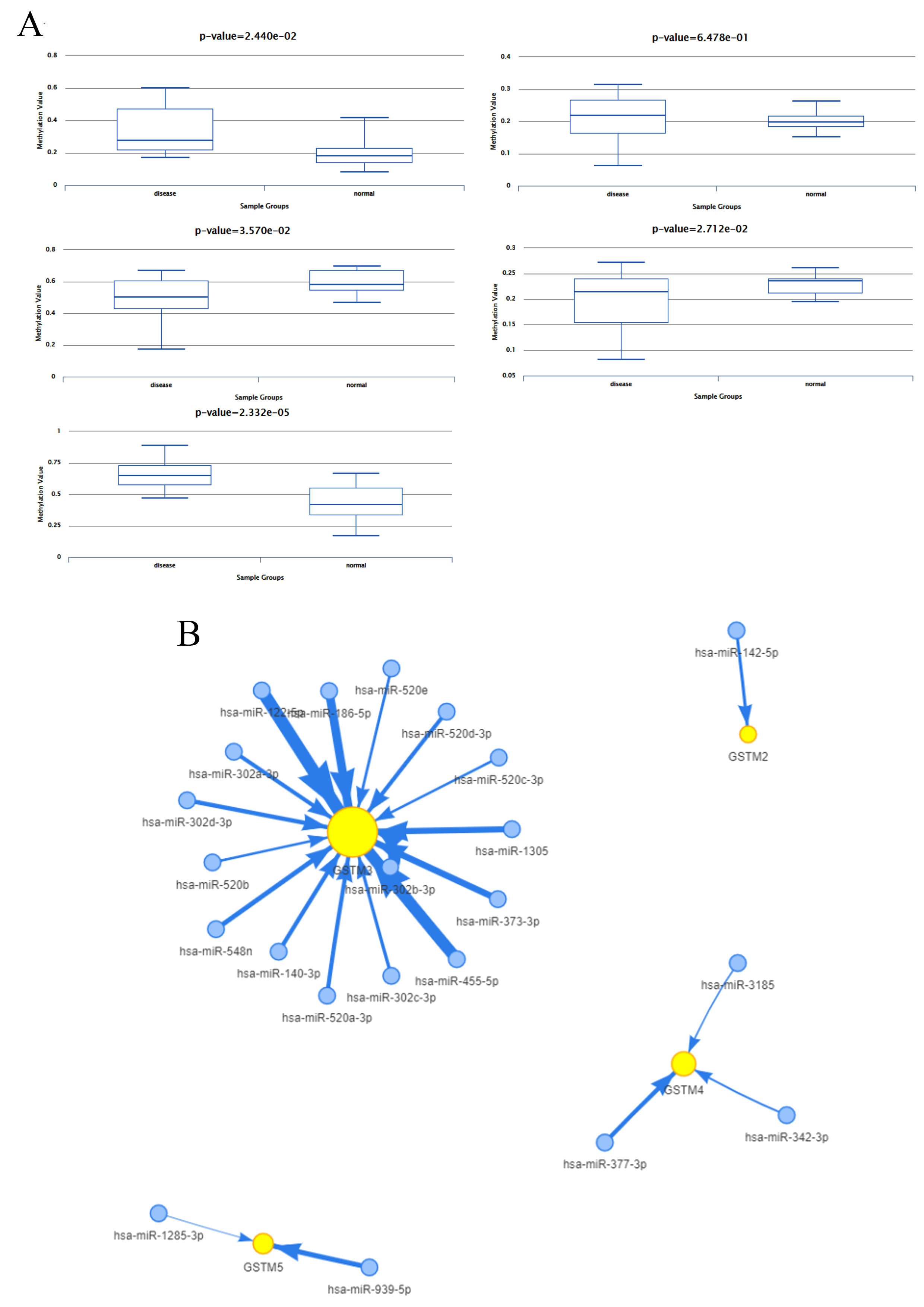

Supplement: Supplementary Figure 1 — The regulatory mechanisms of GSTM members. (A) The DNA m5C methylation level of GSTM1-5 in OC patients and normal women. (B) The miRNA network of GSTMs based on GSCALite database. [file Image_1.jpeg]

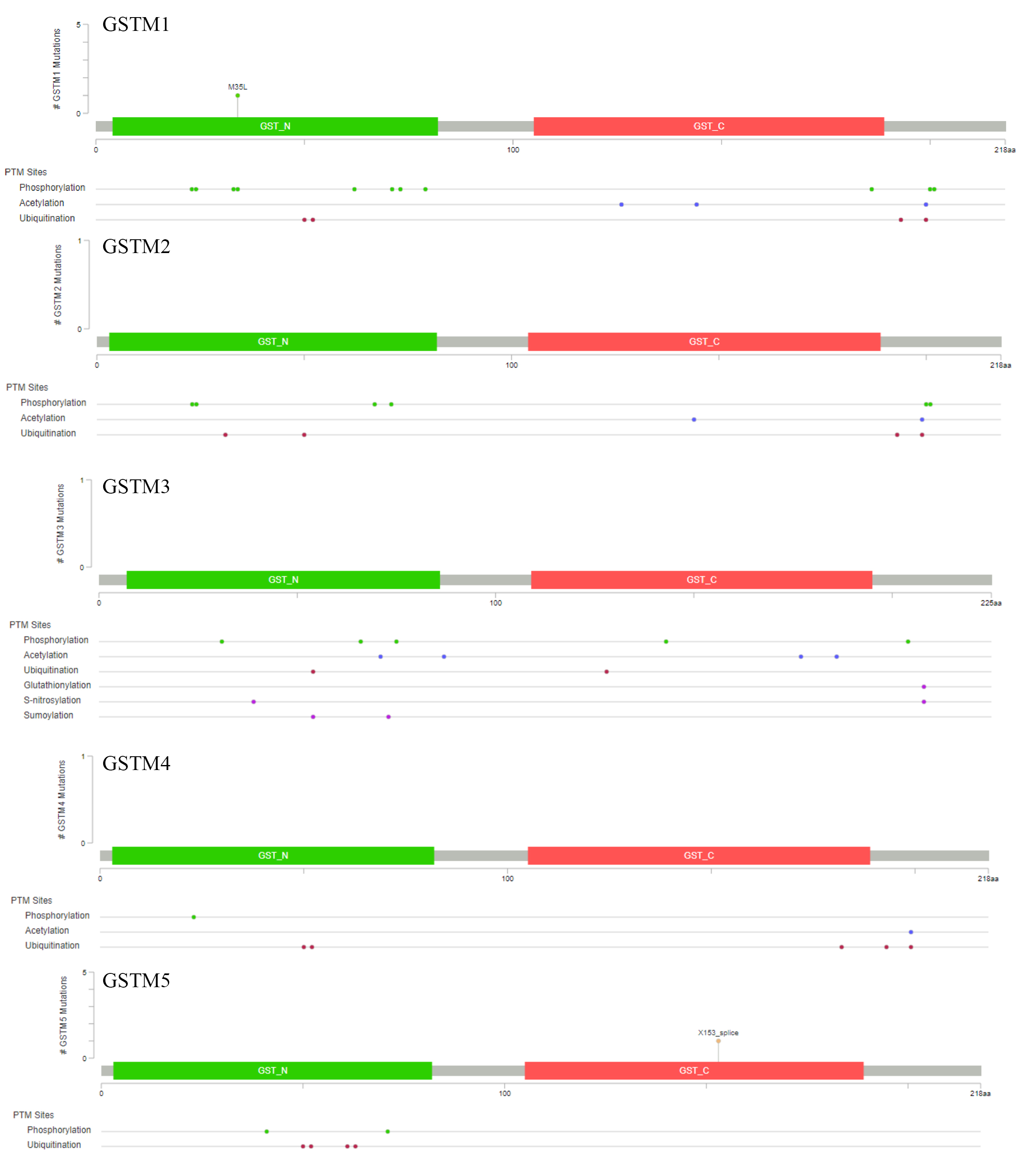

Supplement: Supplementary Figure 2 — The protein structure of GSTM members. [file Image_2.jpeg]

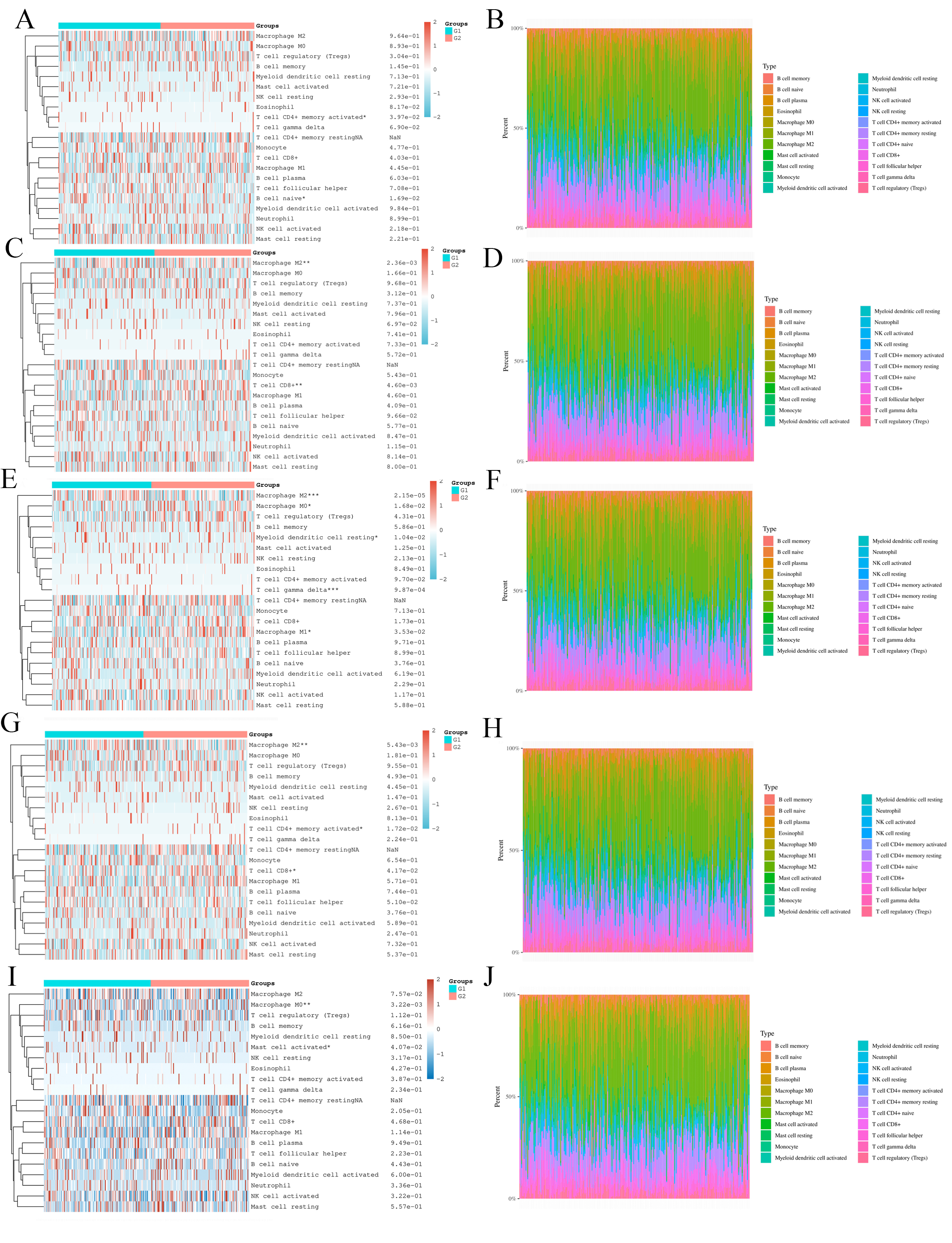

Supplement: Supplementary Figure 3 — The expression distribution of GSTM1-5 immune score in OC samples and normal ovary samples. Immune cell score heatmap for GSTM1 (A), GSTM2 (C), GSTM3 (E), GSTM4 (G) and GSTM5 (I) based on TCGA database via the Wilcox test. The percentage abundance of OC infiltrating immune cells in GSTM1 (B), GSTM2 (D), GSTM3 (F), GSTM4 (H) and GSTM5 (J) group. Different colors indicated different immune cells types. The abscissa represents the OC sample, and the ordinate represents the percentage of immune cell content in each OC sample. *p < 0.05; **p < 0.01; ***p < 0.001. [file Image_3.jpeg]
