# Supplementary material for: Comprehensive analysis of the glutathione S-transferase Mu (GSTM) gene family in ovarian cancer identifies prognostic and expression significance
Source: Front Oncol. 2022 Jul 28;12:968547. doi: 10.3389/fonc.2022.968547 (PMC9366399; doi:10.3389/fonc.2022.968547)
Supplement: Supplementary file 4 [file Table_1.docx]

| **SUPPLEMENTARY TABLE1. The patient's information based on HPA database** | | | | |
| --- | --- | --- | --- | --- |
| Patients ID | Age | Histological classification | Sex | Sample classification |
| 4941 | 41 | Normal tissue | Female | Ovary (T-87000) |
| 4703 | 72 | cystadenocarcinoma, mucinous | Female | Ovary (T-87000) |
| 4401 | 67 | cystadenocarcinoma, serous | Female | Ovary (T-87000) |
| 4395 | 39 | Normal tissue | Female | Ovary (T-87000) |
| 4361 | 70 | cystadenocarcinoma, mucinous | Female | Ovary (T-87000) |
| 4122 | 73 | cystadenocarcinoma, mucinous | Female | Ovary (T-87000) |
| 4030 | 34 | Normal tissue | Female | Ovary (T-87000) |
| 3891 | 44 | Normal tissue | Female | Ovary (T-87000) |
| 3146 | 44 | cystadenocarcinoma, serous | Female | Ovary (T-87000) |
| 3115 | 56 | cystadenocarcinoma, serous | Female | Ovary (T-87000) |
| 2984 | 64 | carcinoma, endometroid | Female | Ovary (T-87000) |
| 2982 | 53 | cystadenocarcinoma, serous | Female | Ovary (T-87000) |
| 2950 | 39 | cystadenocarcinoma, mucinous | Female | Ovary (T-87000) |
| 2889 | 79 | cystadenocarcinoma, serous | Female | Ovary (T-87000) |
| 2724 | 58 | cystadenocarcinoma, serous | Female | Ovary (T-87000) |
| 2713 | 41 | Normal tissue | Female | Ovary (T-87000) |
| 2568 | 62 | carcinoma, endometroid | Female | Ovary (T-87000) |
| 2437 | 59 | cystadenocarcinoma, serous | Female | Ovary (T-87000) |
| 2391 | 69 | cystadenocarcinoma, serous | Female | Ovary (T-87000) |
| 2380 | 39 | Normal tissue | Female | Ovary (T-87000) |
| 2347 | 59 | cystadenocarcinoma, serous | Female | Ovary (T-87000) |
| 2218 | 42 | carcinoma, endometroid | Female | Ovary (T-87000) |
| 2159 | 33 | Normal tissue | Female | Ovary (T-87000) |
| 2114 | 54 | cystadenocarcinoma, serous | Female | Ovary (T-87000) |
| 2082 | 57 | cystadenocarcinoma, serous | Female | Ovary (T-87000) |
| 1911 | 51 | carcinoma, endometroid | Female | Ovary (T-87000) |
| 1844 | 73 | cystadenocarcinoma, mucinous | Female | Ovary (T-87000) |
| 1808 | 51 | Normal tissue | Female | Ovary (T-87000) |
| 1288 | 79 | cystadenocarcinoma, serous | Female | Ovary (T-87000) |
| 925 | 56 | cystadenocarcinoma, serous | Female | Ovary (T-87000) |
| 922 | 51 | cystadenocarcinoma, serous | Female | Ovary (T-87000) |
| 892 | 69 | cystadenocarcinoma, serous | Female | Ovary (T-87000) |
| 857 | 37 | cystadenocarcinoma, mucinous | Female | Ovary (T-87000) |
| 610 | 70 | carcinoma, endometroid | Female | Ovary (T-87000) |
| 592 | 71 | cystadenocarcinoma, serous | Female | Ovary (T-87000) |
| 565 | 70 | carcinoma, endometroid | Female | Ovary (T-87000) |
